# Analysis of expression profiles of long noncoding RNAs and mRNAs in brains of mice infected by rabies virus by RNA sequencing

**DOI:** 10.1038/s41598-018-30359-z

**Published:** 2018-08-08

**Authors:** Pingsen Zhao, Sudong Liu, Zhixiong Zhong, Tianqi Jiang, Ruiqiang Weng, Mengze Xie, Songtao Yang, Xianzhu Xia

**Affiliations:** 1 Clinical Core Laboratory, Meizhou People’s Hospital (Huangtang Hospital), Meizhou Hospital Affiliated to Sun Yat-sen University, Meizhou, 514031 P. R. China; 2 Center for Precision Medicine, Meizhou People’s Hospital (Huangtang Hospital), Meizhou Hospital Affiliated to Sun Yat-sen University, Meizhou, 514031 P. R. China; 3 Guangdong Provincial Engineering and Technology Research Center for Molecular Diagnostics of Cardiovascular Diseases, Meizhou, 514031 P. R. China; 4 Meizhou Municipal Engineering and Technology Research Center for Molecular Diagnostics of Cardiovascular Diseases, Meizhou, 514031 P. R. China; 5 Meizhou Municipal Engineering and Technology Research Center for Molecular Diagnostics of Major Genetic Disorders, Meizhou, 514031 P. R. China; 6 College of Veterinary Medicine, Northeast Agricultural University, Harbin, 150030 China; 7 College of Veterinary Medicine, Jilin University, Changchun, 130062 China; 8 Institute of Military Veterinary, Academy of Military Medical Sciences, Changchun, 130122 China

## Abstract

Rabies, caused by rabies virus (RABV), is still the deadliest infectious disease. Mechanism of host immune response upon RABV infection is not yet fully understood. Accumulating evidences suggest that long noncoding RNAs (lncRNAs) plays key roles in host antiviral responses. However, expression profile and function of lncRNAs in RABV infection remain unclear. In the present study, expression profile of lncRNAs and mRNAs profiles were investigated in RABV-infected brain tissues of mice by RNA sequencing. A total of 140 lncRNAs and 3,807 mRNAs were differentially expressed in RABV-infected animals. The functional annotation and enrichment analysis using Gene Oncology (GO) and Kyoto Encyclopedia of Genes and Genomes (KEGG) revealed that differentially expressed transcripts were predominantly involved in signaling pathways related to host immune response. The expression profiles of the selected lncRNAs in brains of mice during RABV infections were verified by quantitative real time polymerase chain reaction (qRT-PCR). To our knowledge, this is the first report to profile the lncRNA expression in RABV infected mice. Our findings provide insights into understanding the role of lncRNAs in host immune response against RABV infection.

## Introduction

Rabies is one of the deadliest zoonosis disease caused by rabies virus (RABV)^1^. It is nearly 100% fatal once clinical symptoms develop^2^. Rabies claims more than 60,000 human deaths annually, which is more than any other single zoonotic disease in the world. More than 80% of the deaths occurred in countries in Asia. China is the second most burden countries in the world. It showed that 40% of the deaths are children and 99% of the cases are resulted from bites of infected dogs^3^. Meanwhile, in developed countries like USA and Canada, bat RABV poses a serious threat to public health^4^.

RABV is a negative-stranded RNA virus that belongs to the family *Rhabdoviridae*, genus *Lyssavirus*, and species *Rabies lyssavirus*. Genome of RABV is approximately 12 kb and encodes five structural proteins, i.e. nucleoprotein (N), phosphoprotein (P), matrix protein (M), glycoprotein (G) and RNA-dependent RNA polymerase (L)^5^. Most RABV infections start from a dermal or muscular wound. RABV replicates locally in muscle tissue and then enters a neuron and spreads to motor neurons through synapses between muscles and motor neurons. It transports to central neural system (CNS) by retrograde axonal transport. Displaying of clinical symptoms means RABV reached the CNS^6^, where RABV elicit neuronal dysfunction and ultimately lead to death^7^.

Interferon (IFN)-mediated immune response is essential for protection against RABV infection^8^. Studies have shown that IFN-stimulated genes (ISGs), which were the effector of type I IFN response, exerted diverse antiviral effects^9,10^. Previous studies have demonstrated that deficiency in IFN production increased susceptibility to RABV in mouse model^11^. Although much advances have been achieved in prevention of RABV, the mechanism by which RABV causes fatal disease remains unclear.

Long noncoding RNAs (lncRNAs) are transcripts longer than 200 nucleotides and incapable of coding functional proteins. Most lncRNAs are capped at the 5′-end and polyadenylated at the 3′-end^12^. According to their genomic position, lncRNAs are generally classified as intergenic, intronic, bidirectional, antisense and pseudogene^13^. In the recent years, increasing evidences suggested that lncRNAs regulated numerous physiological processes, such as differentiation^14^, apoptosis^15^, development^16^, and immune responses^17^. In 2006, Rangarajan *et al*.^18^ first reported a virus-induced lncRNA (VINC) in the CNS of mouse after Japanese encephalitis infection. Since then, many viral infections such as influenza (IAV)^19^, HIV^20^, hepatitis B^21^ were reported to induce specific lncRNAs. LncRNA NRAV is downregulated during IAV infection and negatively regulates the transcription of ISGs^22^. Meanwhile, NRAV is the first lncRNA that is involved in inhibiting HIV-1 replication and facilitates the expression of antiviral genes during influenza virus and herpes simplex virus infection^23^. However, little is known about lncRNA expression profile and their regulating roles in immune responses during RABV infection.

To explore the role of lncRNAs during RABV infection, we analyzed the lncRNA expression profile in brain tissues of mice infected by RABV strain CVS-11 utilizing RNA sequencing (RNA-Seq). Our results indicated that RABV induced significant changes in lncRNA expression. Gene ontology (GO) and Kyoto Encyclopedia of Genes and Genomes (KEGG) analysis revealed that differentially expressed lncRNAs regulated immune response against RABV infection. To our knowledge, this is the first study to report profile the lncRNA expression in RABV infected mice. Our findings provide insights into understanding the role of lncRNAs in host immune response against RABV infection.

## Results

### RNA-seq and identification of differentially expressed lncRNA

To investigate lncRNA expression profile in mice infected with RABV, high-throughput RNA sequencing was performed on CVS11 infected brain tissues of mice. We sequenced 15 rRNA-deprived total RNA samples, including 5 brain tissues of mock-infected mice and 10 brain tissues of CVS-11 infected of mice. Each assay was duplicates. Average 80 million raw reads were produced for each sample using Illumina HiSeq platform by two-pair end sequencing. After removing the low-quality and adaptor sequences, clean reads were further analyzed.

**Figure 1:**
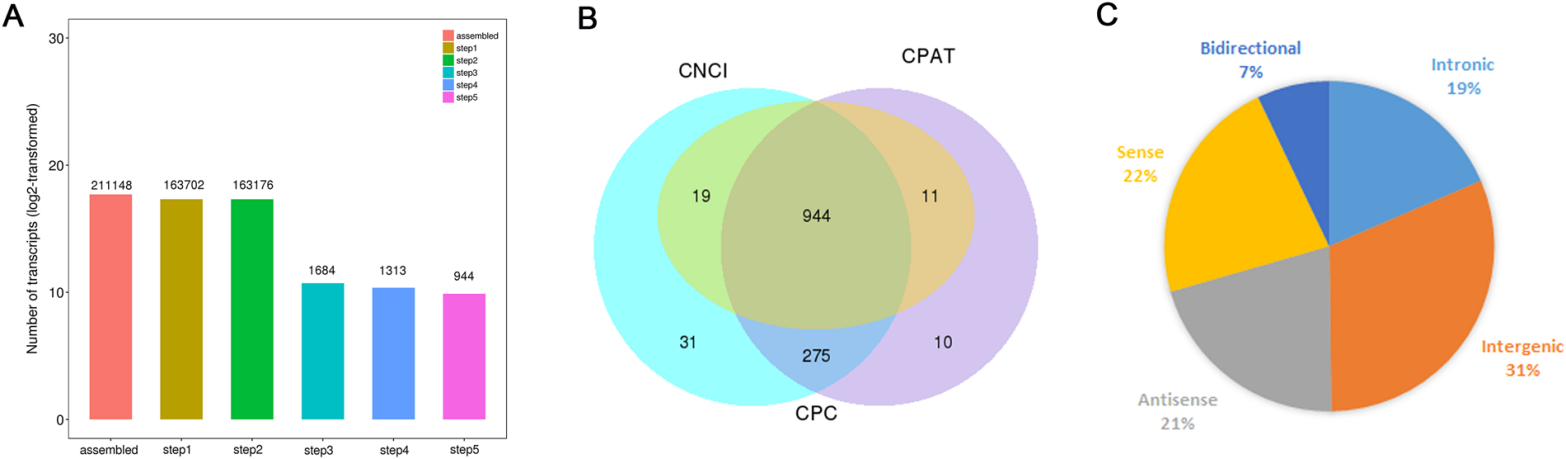
Identification of novel lncRNAs in brain tissues of mice after RABV infection. (**A**) Screen of lncRNAs in RABV infected brain tissues of mice. (**B**) Evaluating the coding capacity of assembled transcripts using CNCI, CPC and CPAT. (**C**) Classification of lncRNAs based on genomic location.

Based on the specific structure and non-coding characteristics of lncRNAs, transcripts were scanned by 5 steps to identify the annotated and novel lncRNAs. 944 novel lncRNAs were assembled by Cuffilinks (Fig. 1A). The coding capacity of transcripts were evaluated by three tools, i.e. Coding-Non-Coding-Index (CNCI), Coding Potential Calculator (CPC) and coding-potential assessment tool(CPAT) (Fig. 1B). Meanwhile, based on the relative genomic locations to coding genes, the lncRNAs identified were divided into five classifications including intergenic lncRNA (31%), intronic lncRNA (19%), antisense lncRNA (21%), sense lncRNA (22%) and bidirectional lncRNA (7%) (Fig. 1C).

**Figure 2:**
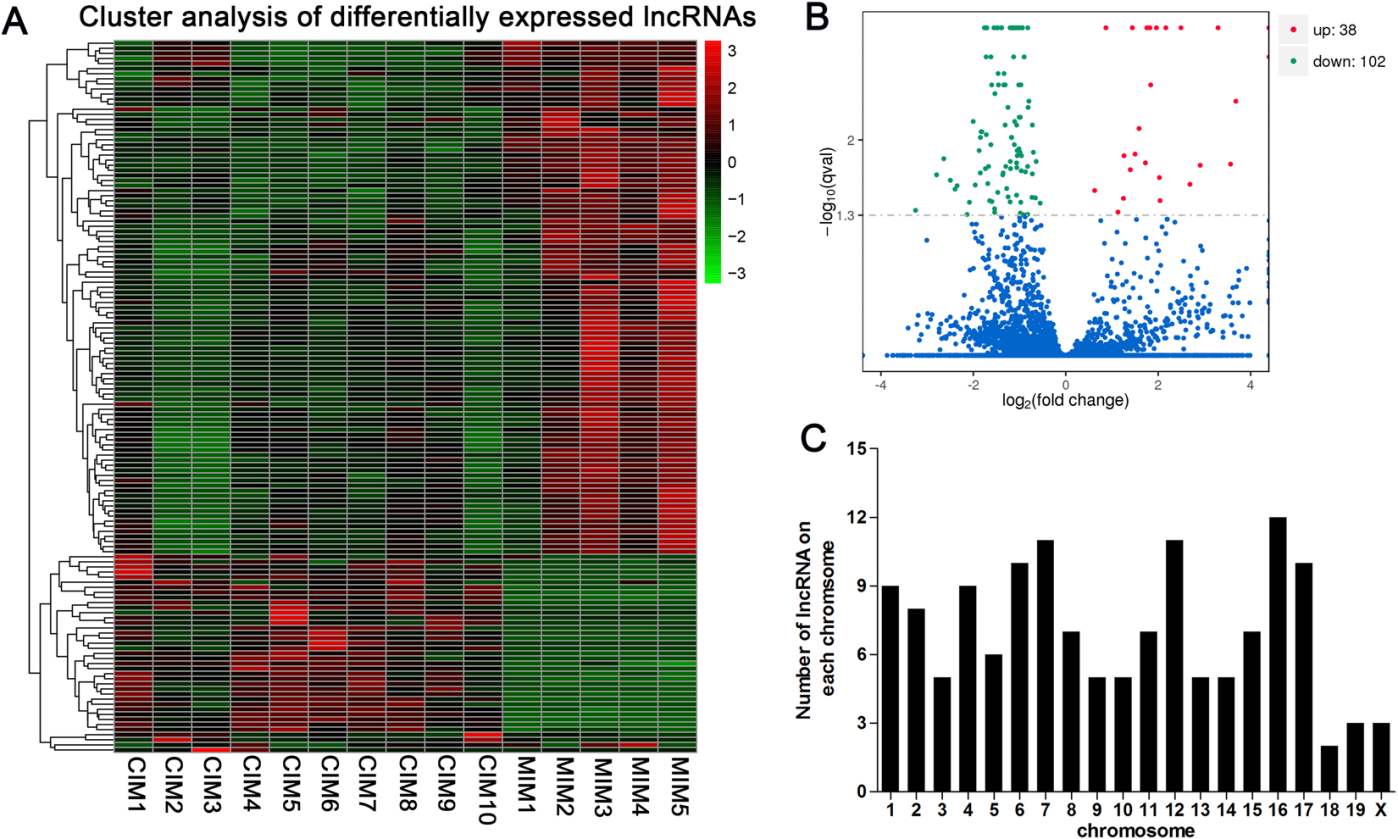
The expression profile of lncRNAs in brain tissues of mice. (**A**) Hierarchical clustering of differentially expressed lncRNAs. (**B**) Volcano plot of differentially expressed lncRNAs in RABV infected brain tissues of mice compared with mock infected controls. (**C**) Distribution of differentially expressed lncRNAs in each chromosome.

Hierarchical clustering was used to analyze the lncRNA expression profiles in mock- or RABV-infected mice. As it was observed, the lncRNA expression profiles were significantly modified after RABV infection (Fig. 2A). A total of 140 lncRNAs were differentially expressed in mice at days post infection (dpi) 8, with 38 lncRNAs up-regulated and 102 lncRNAs down-regulated (Fig. 2B). Of the dysregulated lncRNAs, 20 lncRNAs were changed with a fold change (FC) of more than 5.0, compared with mock infected group (Table 1). The most up-regulated lncRNA was AW112010, with a FC of more than 140, and the most down-regulated transcript was a novel lncRNA, termed LNC_000415 with a FC of more than 9 (Table 1).

**Table 1:** The top 20 differentially expressed lncRNAs in RABV infected mice.

lncRNA ID	Ensembl	Locus	Regulation	Fold change	q value
AW112010	ENSMUST00000099676	19:11047616–11050566	Up	141.25	0.00091
AU020206	ENSMUST00000181224	7:75769761–75782099	Up	58.46	0.00091
AI662270	ENSMUST00000143673	11:83223575–83226604	Up	40.52	0.00091
Ifi30	ENSMUST00000222087	8:70762773–70766663	Up	32.01	0.00091
Gm20559	ENSMUST00000201831	6:3333193–3346071	Up	23.93	0.00091
BC018473	ENSMUST00000156293	11:116752166–116759373	Up	16.60	0.014012
LNC_000406	—	17:29430267–29437310	Up	16.52	0.005568
H19	ENSMUST00000136359	7:142575528–142578143	Up	12.83	0.004372
LNC_000104	—	11:63619195–63620383	Up	11.85	0.016781
Gm12840	ENSMUST00000156081	4:117700187–117700923	Up	9.82	0.00091
LNC_000415	—	17:66233506–66266999	Down	9.52	0.045051
3930402G23Rik	ENSMUST00000040608	8:10924426–10928696	Up	7.51	0.017203
2810407A14Rik	ENSMUST00000189929	16:87787571–87839293	Down	6.94	0.021077
F630028O10Rik	ENSMUST00000147681	X:96239925–96243636	Up	6.44	0.025813
LNC_000019	—	1:77522264–77560578	Down	6.23	0.014936
LNC_000745	—	6:95905307–95922964	Down	5.65	0.023704
Gm7932	ENSMUST00000205047	6:48860328–48866083	Up	5.63	0.00091
Gm31518	ENSMUST00000211925	8:95593421–95613932	Down	5.27	0.028657
LNC_000035	—	1:30120172–30122900	Down	5.10	0.026609

The differentially expressed lncRNA in RABV-infected mice were widely scattered in all chromosomes, while the numbers were various in different chromosomes. Chromosome 7, 12 and 16 had the largest number of altered lncRNAs, while 18,19 and x had the least altered lncRNAs (Fig. 1C).

### Differential expression of mRNAs in brain tissues of mice between mock- and RABV-infected groups

**Figure 3:**
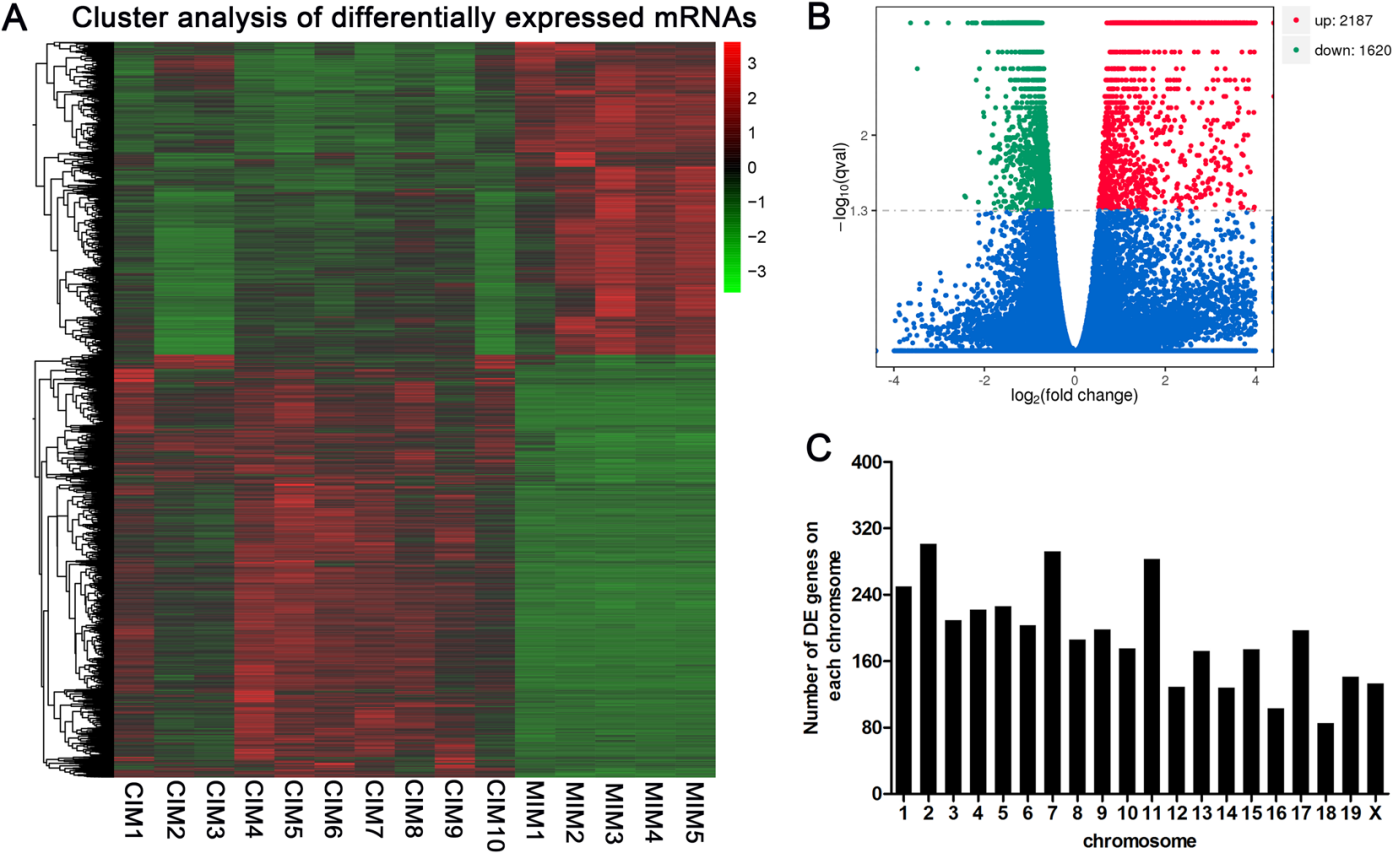
The expression profile of mRNAs in brain tissues of mice (**A**) Hierarchical clustering of differentially expressed mRNAs. (**B**) Volcano plot of differentially expressed mRNAs in RABV infected brain tissues of mice compared with mock infected controls. (**C**) Distribution of differentially expressed lncRNA in each chromosome.

We also examined the change of mRNA expression in brain tissues of mice post CVS-11 infection. Hierarchical cluster analysis showed that mRNA expression profile was significantly changed in mice after RABV infection compared with mock-infected controls (Fig. 3A). A total of 3,807 lncRNAs were differentially expressed in the CVS-11 infected mice (FC ≥ 2 and *P* < 0.05), including 2,187 up-regulated and 1,620 down-regulated (Fig. 3B). To our surprise, 67 genes were upregulated with a fold change of more than 100 after infection. The most up-regulated gene was Cyba (FC = 4.75E + 30). The most down-regulated genes in our study are Alb, with a fold change of 31.16. The top 20 differentially expressed genes were listed in Table 2.

**Table 2:** The top 20 differentially expressed mRNAs in RABV infected mice.

Gene symbol	Ensembl ID	Locus	Regulation	Fold change	q value
Cyba	ENSMUST00000212600	8:119910359–124345722	Up	4.74E + 30	0.027449
Lif	ENSMUST00000066283	11:4257556–4272514	Up	33775.75	0.042156
Ifit1bl2	ENSMUST00000087357	19:34617048–34640743	Up	8902.53	0.036319
Txk	ENSMUST00000113604	5:72695977–72752777	Up	4014.77	0.033761
Oas3	ENSMUST00000044833	5:120753097–120777661	Up	816.25	0.022834
H2-Q6	ENSMUST00000174699	17:35424849–35430055	Up	739.78	0.008388
Irf7	ENSMUST00000026571	7:141228788–141266481	Up	607.47	0.016781
Fcgr4	ENSMUST00000078825	1:171018919–171029761	Up	602.56	0.018905
Ly6c2	ENSMUST00000100542	15:75108160–75111970	Up	596.57	0.004372
H2-Q7	ENSMUST00000116598	17:35439154–35443773	Up	566.59	0.049174
Ifi47	ENSMUST00000046704	11:48904655–49135387	Up	544.35	0.036963
Gbp10	ENSMUST00000065588	5:105214906–105293696	Up	512.67	0.042532
Nlrc5	ENSMUST00000211816	8:94422897–94527272	Up	486.41	0.029481
Iigp1	ENSMUST00000066912	18:60376028–60392627	Up	454.09	0.00091
F830016B08Rik	ENSMUST00000171297	18:60293379–60303016	Up	366.58	0.00091
Lcn2	ENSMUST00000050785	2:32384632–32388252	Up	351.27	0.00091
Serpina3g	ENSMUST00000043315	12:104236251–104241939	Up	347.72	0.037618
Ifi204	ENSMUST00000111214	1:173747292–173766919	Up	347.23	0.00091
Igtp	ENSMUST00000035266	11:58199555–58222782	Up	336.08	0.00091
Ifi209	ENSMUST00000056071	1:173630916–173647928	Up	335.73	0.003719

Similar to the distribution pattern of lncRNAs, the differentially expressed mRNAs in RABV infected mice were not equally scattered among chromosomes. The chromosome 2, 7 and 11 had the most differentially expressed mRNAs and chromosome 16 and 18 have the least numbers, while Y chromosome was absent of differentially expressed mRNAs (Fig. 3C).

### Genomic features of lncRNAs and mRNAs in mice

**Figure 4:**
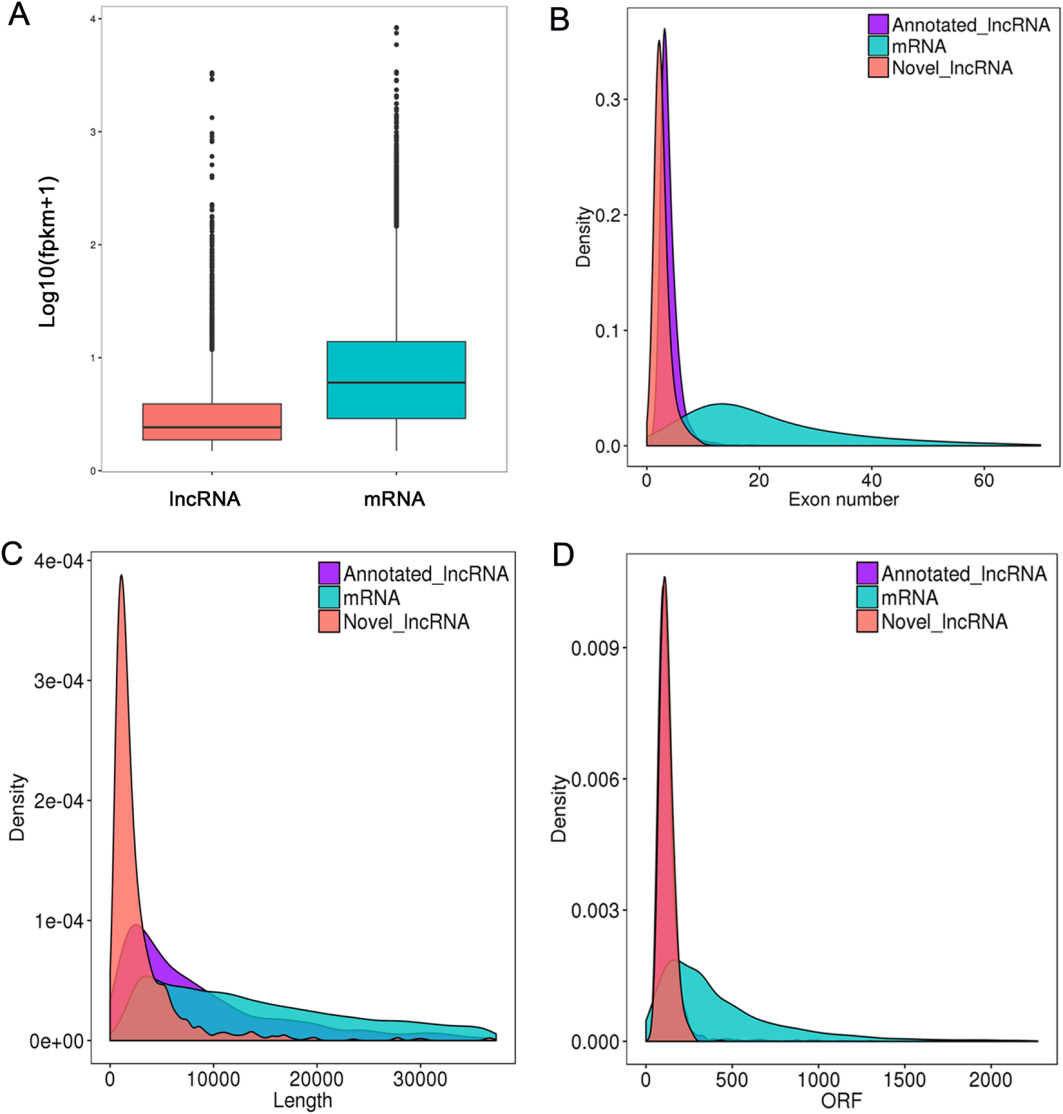
Genomic features of lncRNAs and mRNAs in RABV infected brain tissues of mice. (**A**) Comparison of lncRNAs and mRNAs expression level. (**B**) Comparison of exon number between lncRNA and mRNAs (**C**) Length distribution of lncRNAs and mRNAs. (**D**) Length of ORFs between lncRNAs and mRNAs.

Then we systematically analyzed the basic features of the lncRNAs and compared them with protein-coding genes. As shown in Fig. 4A, the average expression levels of lncRNAs were lower than those of mRNAs. The length of transcripts of lncRNAs was shorter than those of mRNAs (Fig. 4B). Moreover, the exon number of lncRNA was also less than those of mRNAs (Fig. 4C). Furthermore, most of the mRNAs had a longer Open Reading Frames (ORFs) than those of lncRNAs (Fig. 4D).

### Functional prediction of RABV-induced lncRNAs

**Figure 5:**
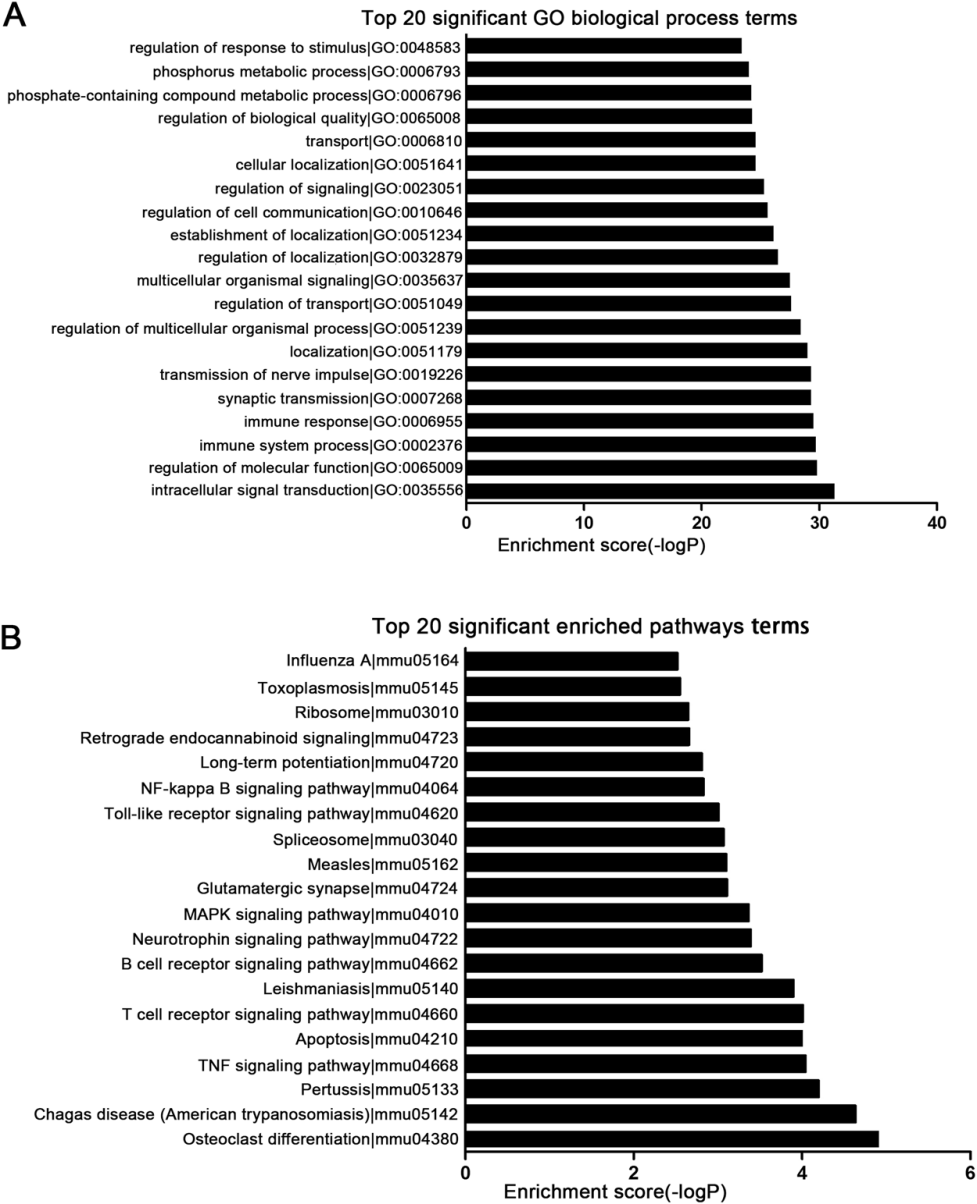
Go enrichment and KEGG pathway analysis of target genes of differentially expressed lncRNAs. (**A**) Top 20 GO biological processes enriched among target genes of differentially expressed lncRNAs. (**B**) The top 20 pathways enriched among target genes of differentially expressed lncRNAs.

To better understand the functions of differentially expressed lncRNAs in RABV infected mice, GO term and KEGG pathway analysis was performed to predict the functions of *cis-* and *trans-* target genes of differentially expressed lncRNAs. We found that the target genes of differentially expressed lncRNAs were highly enriched in biological processes like Intracellular signal transduction, Immune response and Synaptic transmission. The top 20 significant GO biological terms were presented in Fig. 5A. The targets of differentially expressed lncRNAs were involved in important signaling pathways, such as TNF signaling pathway, Toll-like receptor signaling pathway, NF-κB signaling pathway and MAPK signaling pathway. The top 20 significant enriched pathways were presented in Fig. 5B. These findings suggested that lncRNAs regulate the immune responses during RABV infection.

### Validation of differentially expressed lncRNAs by quantitative PCR

**Figure 6:**
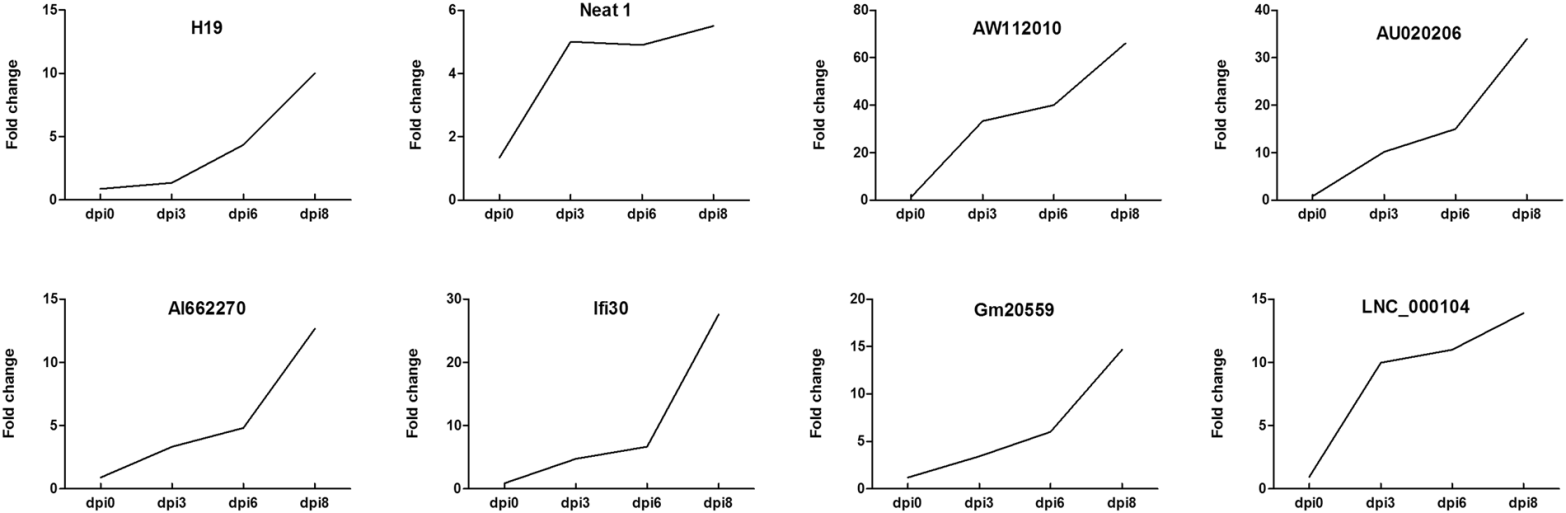
Expression patterns of selected differentially expressed lncRNAs on different time points post RABV infection. Mice were infected with CVS-11 or equal volume of DMEM, and brain samples were collected at dpi 0, 3, 6 and 8 for analysis of selected lncRNAs by qRT-PCR.

RNA-seq analysis indicated that 140 lncRNA were differentially expressed post RABV infection. To validate the RNA-seq data, we investigated the expression levels of the eight most up-regulated lncRNAs at four time points after RABV infection, i.e. dpi 0, 3, 6 and 8, using quantitative real-time PCR (qRT-PCR). The results showed that expression patterns of these eight selected lncRNAs were consistent with RNA-seq data (Fig. 6). Moreover, the levels of these lncRNAs continuously increased from dpi 3 to dpi 8, which may reflect their correlations with progression of clinical symptom.

## Discussion

RABV is still one of the deadliest zoonoses and remains as an important threat to public health in the world. Currently, although rabies is prevented by giving post-exposure prophylaxis (PEP) promptly, it lacks curable treatment. Effective protection of exposed subjects of rabies correlates with the induction of rabies-specific virus-neutralizing antibodies (VNAs). However, current vaccine not only requires multiple injections but also time-consuming and expensive, thus prevent many rabies exposed subjects away from timely vaccinated. As a result, rabies still cause around 70,000 deaths annually around the world despite efficacious vaccines are available^24^. Therefore, it is an emergent need to develop a cost-effective vaccine which elicits long-lasting immunity by a single vaccination and could ideally clear virus infection from the CNS.

During infection, virus is detected by pattern-recognition receptors (PPRs), either canonical or non-canonical, which activate nuclear factor κB (NF-κB) and interferon regulatory transcription factors (IRFs) and induce the expression of type I interferons^25,26^. When RABV infections occur, the innate immune responses are promptly induced. PRRs are activated in the periphery and RABV is recognized in the CNS by retinoic-acid-inducible gene I (RIG-I), which sequentially activate NF-κB and type I IFN-regulated responses^27,28^. However, although much advance has been achieved on RABV biology and anti-RABV immune response, the mechanism underlies how RABV causes fatal disease is not fully understood. Previously, we found that protein-coding gene profile of host cell was significantly changed after RABV infection. We have identified some genes that function against viral replication, i.e. interferon-stimulated genes 15 (ISG15) and ubiquitin-like modifier-activating enzyme 7(UBA7)^29,30^. Recently, many studies have suggested that lncRNAs played key roles in the host immune response against viral infections^31,32^. However, the role of lncRNAs in RABV infection remained unclear. In the present study, we examined the expression profile of lncRNAs and mRNAs in brain tissues of mice after RABV infection. We identified 140 lncRNAs that were significantly differentially expressed between mock- or RABV-infected mice. To be noted, several lncRNAs, i.e. AW112010, AU020206, AI662270 and Ifi30 were up-regulated with a fold change of more than 30. The expression of lncRNAs has been confirmed by qRT-PCR. The dynamic change of lncRNA expression in brain tissues of mice further suggested that lncRNAs might play significant biological roles in RABV infection. Meanwhile, our results showed that 3,807 mRNAs were differentially expressed after infected with CVS-11, including 2,187 up-regulated and 1,620 down-regulated. We also characterized the genomic feature of lncRNAs in brain tissues of mice. Compared with mRNAs, lncRNAs are less enriched in expression, shorter in length, have fewer exons^33–35^. Previous studies have demonstrated that lncRNAs have poor primary sequence conservation compared to protein-coding genes^36^. It has been reported that less than 6% of zebrafish lncRNAs exhibited sequence conservation with lncRNAs of human or mouse and the sequence conservation of lncRNAs between human and other species were only about 12%^37,38^. We also evaluated the sequence conservation of the differentially expressed lncRNAs identified from RABV-infected mouse and found that only about 15% of the lncRNAs appeared to be conserved in human.

Unlike protein-coding genes or microRNAs, the sequences or structures of lncRNAs were currently uninformative for predicting its function^39^. In the present study, the function of lncRNAs was predicted according to their *cis-* or *trans-* target genes. GO terms were significantly enriched in biological processes like Intracellular signal transduction, Regulation of molecular function, Immune system process, Synaptic transmission. It suggested that lncRNAs induced by RABV infection may regulate the immune responses against RABV. KEGG pathway analysis showed that target genes of differentially expressed lncRNA were enriched in the pathways like NF-κB signaling pathway, Toll-like receptor signaling pathway, T cell receptor signaling pathway and TNF signaling pathway, which suggested that lncRNAs take part in host immune response against virus infection through various pathways.

In conclusion, the present study is for the first time to report the expression profile of lncRNAs upon RABV infection in mice. The results suggested that lncRNAs might have key roles in regulating immune responses post RABV infection and exert important biological effects.

## Methods

### Virus

The RABV strain challenge virus standard (CVS-11) was kindly provided by Military Veterinary Institute, Academy of Military Medical Sciences (Changchun, China). Mouse neuroblastoma (NA) cells was seeded in 6-well-plate with a concentration of 4 * 10^5^ in Dulbecco’s modified eagle medium (DMEM) containing 10% fetal bovine serum (FBS), 100U of penicillin/ml, and 100 mg of streptomycin/ml at 37 °C. CVS-11 was added with a MOI of 0.1. The virus was amplified for 72 h and the supernatant were sowed. The virus titer was determined by plaque formation assay on baby hamster Syrian kidney (BHK-21) cells.

### RABV infection

Six-to-eight-week-old male BALB/c mice were purchased from the Guangdong Medical Laboratory Animal Center. Mice were kept in an animal room with stable temperature and light, freely fed and drink. Mice were randomly assigned to two groups: ten for CVS-11 infected group and another ten for mock infected group. For virus infected group, mice were inoculated intracranially with 200 plaque-forming units (PFU) of CVS-11 in 50 µl DMEM, whereas the mock infected group was injected with equal volume of DMEM. On day 8 post infection, mice infected with CVS-11 showed clinical signs, i.e. disordered movement, hunched back, trembling and shaking. Mice from both groups were euthanized, and brain samples were collected. All animal experiments were performed following the National Institute of Health Guide for the Care and Use of Laboratory Animals, and the experimental protocols were approved by the Ethical Committee of Meizhou People’s Hospital (Huangtang Hospital), Meizhou Hospital Affiliated to Sun Yat-sen University, Guangdong, China. All virus experiments were performed at Biosafety Level 2 laboratory.

### Total RNA extraction

Total RNA was extracted from brain tissues of mice using RNeasy Kit (TianGene, Beijing, China) according to manufacturer’s protocol. The quantity and purity of total RNA were evaluated by Nanodrop 2000. The ratio of A260/A280 should be from 1.8 to 2.0. RNA integrity was analyzed by the Bioanalyzer 2100 system (Agilent Technologies, CA, USA).

### High throughput sequencing

Ribo-Zero rRNA removal kit (Epicentre, Madison, WI, USA) was used to remove ribosomal RNA. RNA libraries were prepared using the rRNA-depleted RNA with NEBNext® Ultra™ Directional RNA Library Prep Kit for Illumina (NEB, USA). Library sequencing was performed on a Illumina HiSeq2000 platform (Illumina Inc., San Diego, CA) in ShenZhen Realomics Inc.

### Bioinformatic analysis

Raw data were filtered by removing the adaptors, low-quality reads and poly-N reads to obtain clean data using the SOAPnuke. The Q20, Q30, and GC information were calculated to evaluate the clean data. Then the filtered reads were mapped to the mice reference genome (version: mus_musculus. GRCm38) by Tophat 2. The transcripts were assembled with the mapped reads by reference annotation based transcripts (BRAT) method using Cufflink^40^.

The assembled transcript was identified as a novel lncRNA if (1) exon number ≥ 2, (2) length > 200 nt, (3) FPKM ≥ 0.5, (4) without coding capacity, (5) don’t overlap with mRNA or annotated lncRNA. Coding ability was predicted using coding-non-coding-index (CNCI), coding potential calculator (CPC) and coding-potential assessment tool (CPAT). The expression analysis was performed using Cuffdiff.

Expressed profile of lncRNAs and mRNAs in brains of mice upon RABV infection were shown in Tables S1 and S2.

### GO and KEGG pathway analyses

To predict the target genes of differentially expressed lncRNAs, *cis-* and *trans-* analyses were performed. The genes located within a 10 kb window upstream or downstream of lncRNAs were classified as the *cis* target genes. The *trans* target genes were predicted on the expression levels of coding genes.

GO enrichment analyses were performed to identify biological processes associated with *cis-* or *trans-* target genes of lncRNA. KEGG was used to analyze the associated pathways of *cis-* or *trans-* target genes of the lncRNAs. A false discovery rate (FDR) was used to correct the *P* values. A corrected *P* value (Q values) <0.05 were considered significant.

### Real-time RT-PCR assay

**Real-time RT-PCR** was performed to detect express of the selected lncRNAs using Luna® Universal One-Step RT-qPCR Kit (New England Biolabs, USA) according to manufacturer’s instructions. Primers used for validation of lncRNA expression were shown in Table 3. The amplify program is as follow: 95 °C for 30 s, 40 cycles (95 °C for 5 s, 60 °C for 30 s, and 72 °C for 30 s). The specificity of the amplified products was evaluated using dissociation curves. Relative expression of lncRNA were normalized to Glyceraldehyde 3-phosphate dehydrogenase (GAPDH) using the 2^−ΔΔCt^ method. The tests were triplicated.

**Table 3:** Primers used for validation of expression of lncRNAs by qRT-PCR.

LncRNAs	Primer sequences (5′-3′)
H19	Forward: GGGTCACAAGACACAGATGGGT
	Reverse: CCAGTTATTGAGGCTCTGGCA
Neat 1	Forward: GCAGGACTAGGTGCGTAGTGGA
	Reverse: GCTATCACCCTGGGCCAGA
AW112010	Forward: AAGTCTTCTGCCATCAAGCCA
	Reverse: CCACTTGAGGTTTCCAGTGTGT
AU020206	Forward: CCTGCAGGCTTGATTTCAGTT
	Reverse: AGGGCGTCTGTCAGCCAAGT
AI662270	Forward: GTGCACCCTAAGGATTTATAGGAA
	Reverse: GCCAAAGTGTAAGCAACCAAGA
Ifi30	Forward: TACCATTTTTGTCCCTTCTGCTTC
	Reverse: ACAGGGACTCATAATACAGGCTGAC
Gm20559	Forward: AGGATCATACAAATGAGTTGTGTGG
	Reverse: CTGTATCTGTAGCTTCGTCTGCAAC
LNC_000104	Forward: TGTCATGTTGATCACTTGACTTCAG
	Reverse: AGTCAAAGACAGATGGATGAGCAG
GAPDH	Forward: TTCAACGGCACAGTCAAGGCA
	Reverse: CCACCACATACTCAGCACCAGC

### Statistical analysis

Data were analyzed using Student’s *t*-test or one-way Analysis of variance (ANOVA) followed by Dunnett’s multiple comparison test (compare all groups to the control group). All data are demonstrated as the means ± S.D. (*P < 0.05, **P < 0.01, ***P < 0.001). For correlation studies, a two-tailed non-parametric Spearman analysis was used. *P* ≤ 0.05 were considered as significant.

## Electronic supplementary material

Supplementary Information

Supplementary Information Table S1

Supplementary Information Table S2
