# Supplementary material for: Analysis of expression profiles of long noncoding RNAs and mRNAs in brains of mice infected by rabies virus by RNA sequencing
Source: Sci Rep. 2018 Aug 8;8:11858. doi: 10.1038/s41598-018-30359-z (PMC6082909; doi:10.1038/s41598-018-30359-z)
Supplement: Supplementary file 1 — Supplementary Information [file 41598_2018_30359_MOESM1_ESM.docx]

Analysis of expression profiles of long noncoding RNAs and mRNAs in brains of mice infected by rabies virus by RNA sequencing

Pingsen Zhao^1,2,3,4,5,*, †^, Sudong Liu^1,2,3,4,5,*^, Zhixiong Zhong^2,*^, Tianqi Jiang^6,*^, Ruiqiang Weng^1,2,3,4,5^, Mengze Xie^7^, Songtao Yang^8^, Xianzhu Xia^8^

^1^Clinical Core Laboratory, Meizhou People's Hospital (Huangtang Hospital), Meizhou Hospital Affiliated to Sun Yat-sen University, Meizhou 514031, P. R. China

^2^Center for Precision Medicine, Meizhou People's Hospital (Huangtang Hospital), Meizhou Hospital Affiliated to Sun Yat-sen University, Meizhou 514031, P. R. China

^3^Guangdong Provincial Engineering and Technology Research Center for Molecular Diagnostics of Cardiovascular Diseases, Meizhou 514031, P. R. China

^4^Meizhou Municipal Engineering and Technology Research Center for Molecular Diagnostics of Cardiovascular Diseases, Meizhou 514031, P. R. China

^5^Meizhou Municipal Engineering and Technology Research Center for Molecular Diagnostics of Major Genetic Disorders, Meizhou 514031, P. R. China

^6^College of Veterinary Medicine, Northeast Agricultural University, Harbin 150030, China

^7^College of Veterinary Medicine, Jilin University, Changchun 130062, China

^8^Institute of Military Veterinary, Academy of Military Medical Sciences, Changchun 130122, China

^*^Contributed equally to this work.

^†^Corresponding author: Pingsen Zhao, Head & Associate Professor, Clinical Core Laboratory, Center for Precision Medicine, Meizhou People's Hospital (Huangtang Hospital), Meizhou Hospital Affiliated to Sun Yat-sen University, Meizhou 514031, P. R. China

Add.: No 63, Huangtang Road, Meijiang District, Meizhou 514031, P. R. China

Tel: +753-2131-591, Fax: +753-2131-592

Email: [zhaopingsen01@163.com](mailto:zhaopingsen01@163.com), [zhaopingsen@hotmail.com](mailto:zhaopingsen@hotmail.com)

**Supplementary information**

**Table S1.** Expressed profile of lncRNAs in brains of mice upon RABV infection.

**Table S2.** Expressed profile of mRNAs in brains of mice upon RABV infection.
